# Association between red and processed meat consumption and colorectal cancer risk: a comprehensive meta-analysis of prospective studies

**DOI:** 10.1007/s11357-025-01646-1

**Published:** 2025-04-10

**Authors:** Zoltan Ungvari, Mónika Fekete, Péter Varga, Andrea Lehoczki, Gyöngyi Munkácsy, János Tibor Fekete, Giampaolo Bianchini, Alberto Ocana, Annamaria Buda, Anna Ungvari, Balázs Győrffy

**Affiliations:** 1 Vascular Cognitive Impairment, Neurodegeneration and Healthy Brain Aging Program, Department of Neurosurgery, University of Oklahoma Health Sciences Center, Oklahoma City, OK USA; 2 Stephenson Cancer Center, University of Oklahoma, Oklahoma City, OK USA; 3 Oklahoma Center for Geroscience and Healthy Brain Aging, University of Oklahoma Health Sciences Center, Oklahoma City, OK USA; 4 Department of Health Promotion Sciences, College of Public Health, University of Oklahoma Health Sciences Center, Oklahoma City, OK USA; 5 International Training Program in Geroscience, Doctoral College, Health Sciences Division/Institute of Preventive Medicine and Public Health, Semmelweis University, Budapest, Hungary; 6 Institute of Preventive Medicine and Public Health, Semmelweis University, Budapest, Hungary; 7 Jozsef Fodor Center for Prevention and Healthy Aging, Semmelweis University, Budapest, Hungary; 8 Doctoral College, Health Sciences Division, Budapest, Hungary; 9 Department of Bioinformatics, Semmelweis University, 1094 Budapest, Hungary; 10 Cancer Biomarker Research Group, Institute of Molecular Life Sciences, HUN-REN Research Centre for Natural Sciences, H- 1117 Budapest, Hungary; 11 Department of Medical Oncology, IRCCS Ospedale San Raffaele, Milan, Italy; 12 Vita-Salute San Raffaele University, Milan, Italy; 13 Experimental Therapeutics in Cancer Unit, Instituto de Investigación Sanitaria San Carlos (IdISSC), and CIBERONC, Madrid, Spain; 14 INTHEOS-CEU-START Laboratory, Facultad de Medicina, Universidad CEU San Pablo, 28668 Boadilla del Monte, Madrid, Spain; 15 Department of Biophysics, Medical School, University of Pecs, H- 7624 Pecs, Hungary

**Keywords:** Red meat, Processed meat, Colorectal cancer, Colon cancer, Rectal cancer, Risk factor, Meta-analysis, Dietary recommendation, Semmelweis Study, Aging, Healthy aging, Diet, Dietary

## Abstract

Increasing evidence suggests that red and processed meat consumption may elevate the risk of colorectal cancer (CRC), yet the magnitude and consistency of this association remain debated. This meta-analysis aims to quantify the relationship between red and processed meat intake and the risk of CRC, colon cancer, and rectal cancer using the most comprehensive set of prospective studies to date. We conducted a comprehensive search in PubMed, Web of Science, Cochrane Library, Embase, and Google Scholar databases from 1990 to November 2024, to identify relevant prospective studies examining red, processed, and total meat consumption in relation to colorectal, colon, and rectal cancer risk. Hazard ratios (HR) and 95% confidence intervals (CI) were extracted for each study and pooled using a random-effects model to account for variability among studies. Statistical evaluation was executed using the online platform MetaAnalysisOnline.com. A total of 60 prospective studies were included. Red meat consumption was associated with a significantly increased risk of colon cancer (HR = 1.22, 95% CI 1.15–1.30), colorectal cancer (HR = 1.15, 95% CI 1.10–1.21), and rectal cancer (HR = 1.22, 95% CI 1.07–1.39). Processed meat consumption showed similar associations with increased risk for colon cancer (HR = 1.13, 95% CI 1.07–1.20), colorectal cancer (HR = 1.21, 95% CI 1.14–1.28), and rectal cancer (HR = 1.17, 95% CI 1.05–1.30). Total meat consumption also correlated with an elevated risk of colon cancer (HR = 1.22, 95% CI 1.11–1.35), colorectal cancer (HR = 1.17, 95% CI 1.12–1.22), and rectal cancer (HR = 1.28, 95% CI 1.10–1.48). This meta-analysis provides robust evidence that high consumption of red and processed meats is significantly associated with an increased risk of colorectal, colon, and rectal cancers. These findings reinforce current dietary recommendations advocating for the limitation of red and processed meat intake as part of cancer prevention strategies.

## Introduction

Colorectal cancer (CRC) is the third most commonly diagnosed cancer worldwide and the second leading cause of cancer-related deaths, accounting for approximately 10% of all cancer cases and 9.4% of global cancer deaths [1–3]. In 2020 alone, 1.9 million new CRC cases and 935,000 deaths were reported, with incidence rates expected to rise due to aging populations and lifestyle changes. Among modifiable risk factors, dietary patterns have been increasingly recognized as significant contributors to CRC risk [4, 5], particularly the consumption of red and processed meats [6].

The International Agency for Research on Cancer (IARC) has classified processed meat as a Group 1 carcinogen (carcinogenic to humans) and red meat as a Group 2 A probable carcinogen, based on epidemiological evidence linking these dietary factors to CRC development [6]. The underlying mechanisms involve the use of preservatives (particularly sodium nitrite/E250) leading to the formation of carcinogenic compounds during grilling and cooking of meats, including heterocyclic amines (HCAs), polycyclic aromatic hydrocarbons (PAHs), and N-nitroso compounds (NOCs), which may contribute to DNA damage, oxidative stress, and inflammation in the colonic mucosa [6].

Despite the biological plausibility of this association, inconsistencies remain across epidemiological studies due to differences in study design, population characteristics, dietary exposure assessments, and adjustments for confounding factors [7, 8]. While some studies suggest that only high levels of red and processed meat intake increase CRC risk, others indicate a linear dose–response relationship, where risk gradually increases with higher consumption levels [9–21]. Additionally, the relative contributions of colon cancer versus rectal cancer in response to meat consumption remain poorly defined, further complicating risk assessments, and dietary recommendations.

Given these discrepancies, an updated and comprehensive meta-analysis of prospective cohort studies is warranted to clarify the strength and consistency of the association between red and processed meat consumption and CRC risk. This study aims to quantify the association between red, processed, and total meat intake and CRC risk and provide evidence-based insights to inform public health recommendations and dietary guidelines for CRC prevention.

## Methods

### Search strategy and selection criteria

We performed an extensive systematic review and meta-analysis to explore the relationship between the consumption of red and processed meats and the risk of developing colorectal cancer (CRC). Relevant studies were identified by searching five electronic databases, including PubMed, Web of Science, Cochrane Library, Embase, and Google Scholar [22–81]. The search covered the period from 1990 to November 1, 2024, and was conducted without language restrictions. We also identified studies from previous meta-analyses [9–11].

**Fig. 1:**
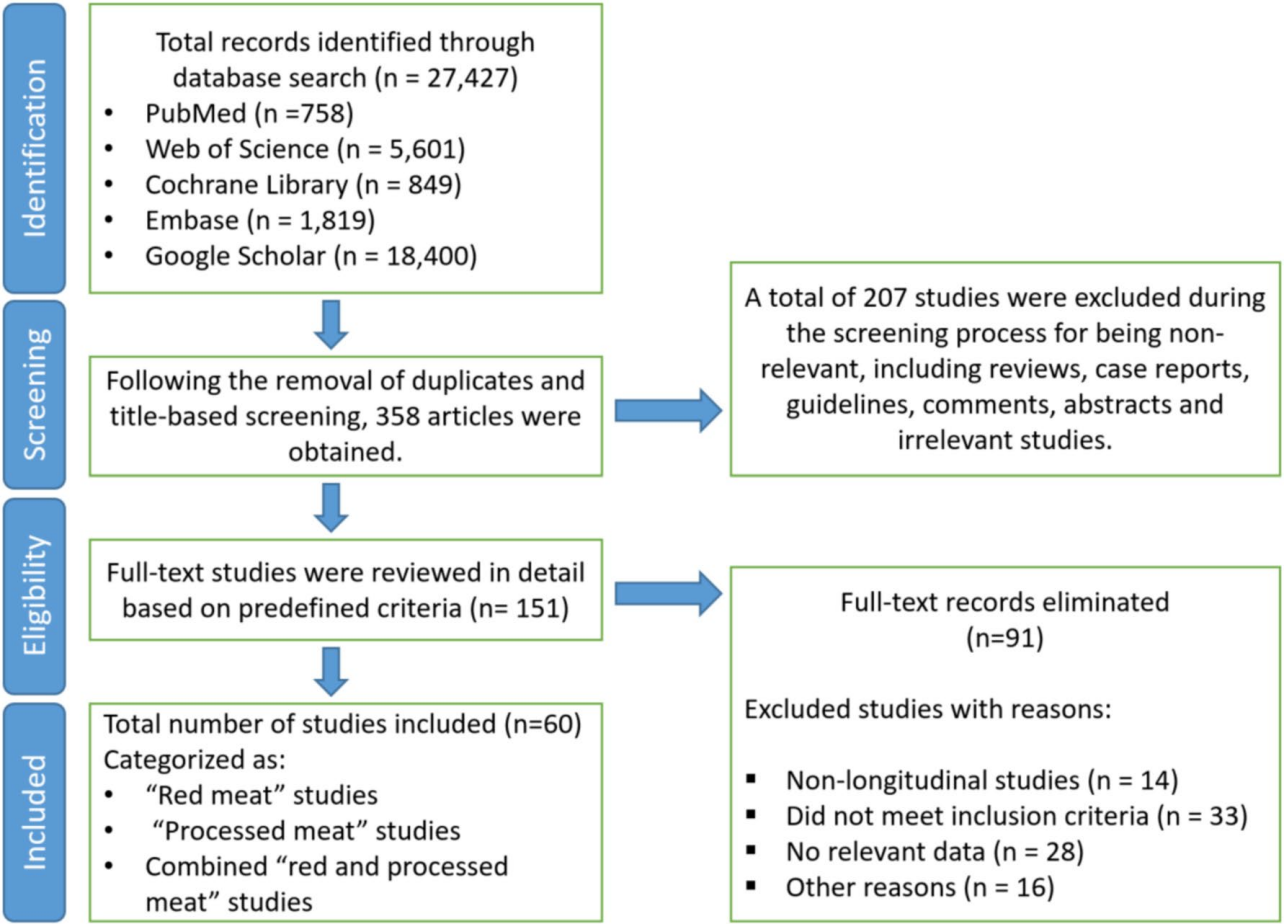
Study selection process for the meta-analysis of prospective studies. After removing duplicates and title-based screening, 358 articles were retained. Following full-text review, 60 studies were included in the meta-analysis including (1) red meat studies, (2) processed meat studies, and (3) combined red and processed meat studies. Exclusions included non-relevant studies (*n* = 207) and full-text articles excluded for reasons such as non-longitudinal design (*n* = 14), failure to meet inclusion criteria (*n* = 33), lack of relevant data (*n* = 28), or other reasons (*n* = 16)

We used search terms such as “red meat,” “processed meat,” “colorectal cancer,” “colon cancer,” and “rectal cancer,” which were combined in various ways to maximize the comprehensiveness of the search. Examples of these combinations included “red meat AND colorectal cancer,” “processed meat AND colon cancer OR rectal cancer,” “red and processed meats AND colorectal cancer,” and “diet AND colorectal cancer risk.” These strategies ensured that we captured all relevant studies addressing the association between the intake of red and processed meats and CRC. Within the selected studies, red meat generally referred to beef, pork, lamb, mutton, and veal, while processed meat encompassed products such as bacon, sausages, ham, and other cured or preserved meats. The combined category “red and processed meats” denoted the intake of both red and processed meats. The details of the study selection process are presented in Fig. 1.

To determine the eligibility of studies, three independent researchers assessed each article against predefined inclusion and exclusion criteria. Any disagreements regarding eligibility were resolved by consensus. The studies included in the analysis examined the relationship between red and processed meat consumption and CRC risk. The diagnosis of CRC in these studies adhered to internationally recognized diagnostic guidelines. Furthermore, the selected studies employed longitudinal designs, assessed meat consumption through self-reports, questionnaires, clinical diagnoses, or other objective methodologies, and reported hazard ratio (HR) estimates along with confidence intervals (CIs) as measures of association. We excluded case reports, commentaries, and conference abstracts, as well as studies that did not specifically investigate the association between red and processed meat consumption and CRC risk. Studies with cross-sectional designs, insufficiently defined CRC diagnoses, or lacking relevant risk data such as HRs or CIs were also excluded.

### Data extraction

Following the selection process, data were extracted independently by three investigators, who cross-verified the extracted information to ensure accuracy. Any discrepancies were resolved through discussion and consensus. The extracted data included the first author’s name, the year of publication, the study design, the total sample size, and the number of CRC cases observed during the follow-up period. For risk estimation, relative risks with their corresponding confidence intervals were collected. When multiple levels of adjustment were available for relative risk estimates, we prioritized the model with the most comprehensive adjustments. This methodological approach enabled a systematic synthesis and meta-analytic evaluation of the relationship between red and processed meat consumption and CRC risk, providing a rigorous and thorough examination of the available evidence.

### Statistical analyses

The statistical evaluation was carried out using the online platform MetaAnalysisOnline.com. To calculate aggregated risk estimates, including hazard ratios (HRs) along with their 95% confidence intervals (CIs), we utilized a random-effects model. This method accommodates differences among studies, thereby improving the applicability of the results to a broader context. Forest plots were constructed to visually depict the findings of individual studies alongside the combined summary estimate, aiding in data interpretation and enabling the detection of heterogeneity between studies.

To quantify inter-study variability, we employed Cochran’s *Q* test and the *I*2 statistic. Cochran’s *Q* test, a chi-squared-based method, was used to determine whether the variability in effect sizes across studies exceeded what could be attributed to random chance. The *I*2 statistic was calculated to measure the percentage of total variation that stemmed from actual differences between studies rather than random noise.

### Evaluation of publication bias

The presence of potential publication bias was examined using funnel plots, which graphically illustrate the relationship between study effect sizes and measures of their precision. Asymmetry in these plots may suggest the presence of bias. Additionally, Egger’s regression analysis was conducted to statistically assess the association between effect sizes and their standard errors, providing a quantitative measure of publication bias.

### Subgroup analyses

We conducted subgroup analyses focusing on distinct cancer types, specifically colon, colorectal, and rectal cancers. For each subgroup, pooled effect estimates were computed alongside heterogeneity metrics to assess the specific impact within each cancer category. Furthermore, analyses were extended to the combined cohort, enabling an evaluation of the aggregated effects across all included conditions.

## Results

### Effect of red meat consumption

**Fig. 2:**
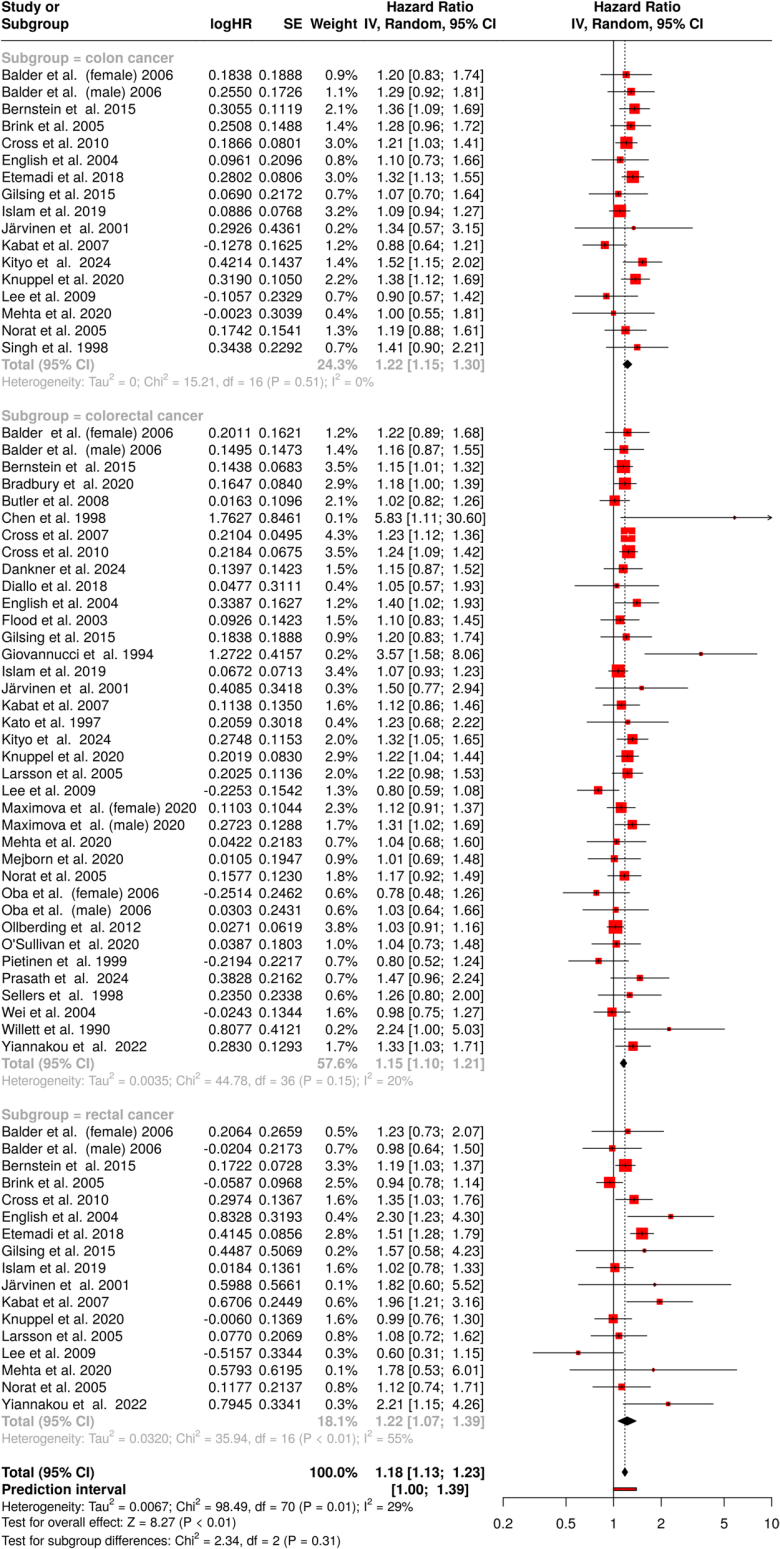
Forest plot of the association between red meat consumption and the risk of colon, colorectal, and rectal cancer. Hazard ratios (HRs) with 95% confidence intervals (CIs) are shown for individual studies and pooled subgroups. Subgroup analyses are presented for colon cancer, colorectal cancer, and rectal cancer, with statistical heterogeneity assessed for each. Squares represent individual study HRs, with the size reflecting the study weight, and diamonds indicate pooled estimates. The overall HR indicates an increased risk of cancer associated with red meat consumption (HR = 1.18, 95% CI 1.13–1.23). The analysis demonstrates moderate heterogeneity across studies (*I*^2^ = 29%; *p* = 0.01). Abbreviations: *CI*, confidence interval; *HR*, risk ratio; *IV*, inverse variance; *SE*, standard error

A total of 71 cohorts were included in the meta-analysis to evaluate the association between red meat consumption and the risk of colon, colorectal, and rectal cancer (Fig. 2). Using a random-effects model and the inverse variance method, the pooled analysis revealed a statistically significant association. The summarized risk estimate indicated an 18% increased risk for individuals with higher red meat consumption compared to those with lower consumption (HR = 1.18; 95% CI 1.13–1.23).

Despite the statistical significance, moderate heterogeneity was observed among the studies, with 29% of the variability attributed to differences in study effects rather than random chance (*I*^2^ = 29%; *p* = 0.01). This heterogeneity suggests that the magnitude and/or direction of the effect varied across the included cohorts. These findings highlight a consistent association between red meat consumption and an elevated risk of colorectal malignancies while underscoring the importance of considering potential variations in study characteristics and populations.

**Fig. 3:**
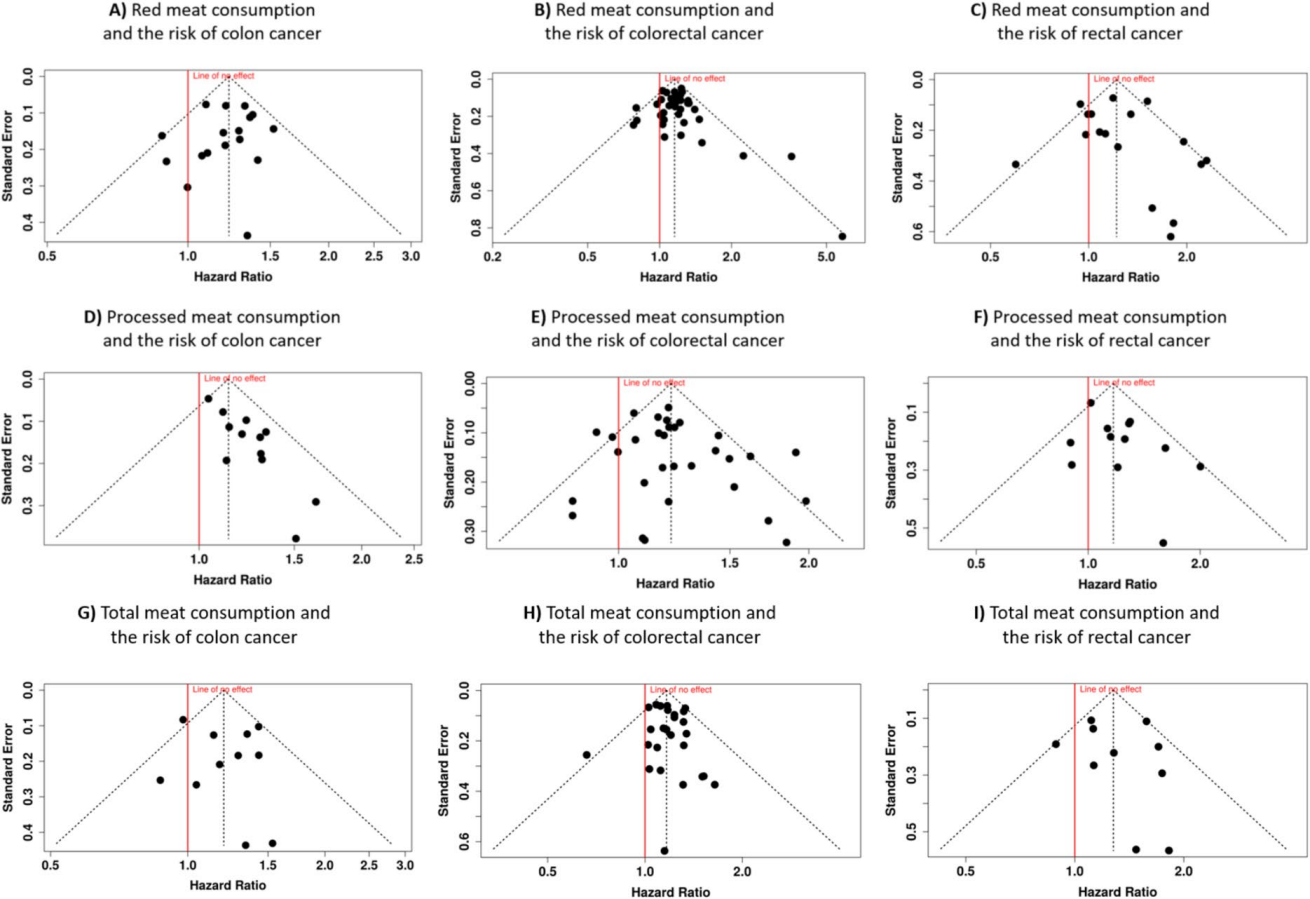
Funnel plots illustrating the relationship between hazard ratios (HRs) and standard error (SE) for the association between red meat (**A–C**), processed meat (**D–F**), or total meat consumption (**G–I**) and the risk of various gastrointestinal cancer subtypes: colon cancer (**A, D**, **G**), colorectal cancer (**B, E, H**), and rectal cancer (**C, F, I**). The shape and symmetry of the funnel plots can offer insights into potential publication bias, with asymmetrical plots indicating the possibility of selective reporting or publication of the studies with certain outcomes

The analysis of publication bias across studies examining the relationship between red meat consumption and cancer risk yielded no evidence of bias for colon, colorectal, or rectal cancer. For colon cancer, the funnel plot did not reveal any asymmetry, and statistical testing using Egger’s test confirmed the absence of significant bias (intercept: − 0.36, 95% CI − 1.49 to 0.77, *t* = − 0.619, *p* = 0.545) (Fig. 3A). Similarly, for colorectal cancer, the funnel plot suggested no signs of publication bias, with Egger’s test further supporting this finding (intercept: 0.3, 95% CI − 0.43 to 1.04, *t* = 0.814, *p* = 0.421) (Fig. 3B). Lastly, in the case of rectal cancer, both the funnel plot and Egger’s test indicated no evidence of asymmetry, suggesting that publication bias did not distort the results (intercept: 0.47, 95% CI − 0.89 to 1.84, *t* = 0.683, *p* = 0.505) (Fig. 3C).

### Effect of processed meat consumption

**Fig. 4:**
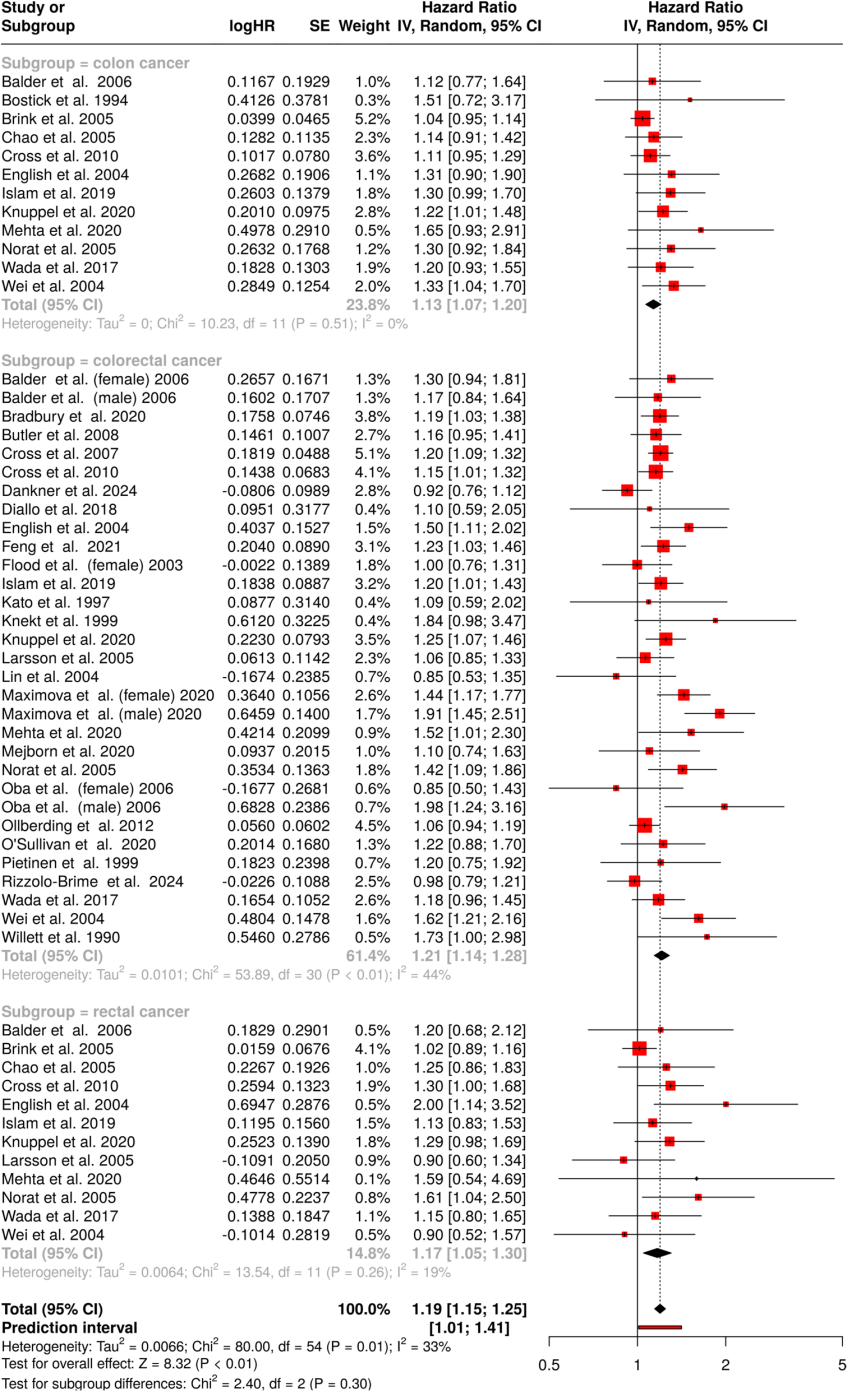
Forest plot of the association between processed meat consumption and the risk of colon, colorectal, and rectal cancer. Each subgroup presents individual study results with hazard ratios (HRs) and 95% confidence intervals (CIs), using a random-effects model. The pooled estimates for colon, colorectal, and rectal cancer are shown, along with heterogeneity statistics. According to the overall HR presented at the bottom of the plot, processed meat consumption is associated with an increased risk of colon, colorectal, and rectal cancer (HR = 1.19, 95% CI 1.15–1.25). Moderate heterogeneity is observed among studies (*I*^2^ = 33%; *p* = 0.01). *Abbreviations: CI*, confidence interval; *HR*, hazard ratio; *IV*, inverse variance; *SE*, standard error

In this comprehensive meta-analysis examining the relationship between processed meat consumption and colon cancer, colorectal, and rectal cancers, data from 55 cohorts were systematically evaluated as depicted in Fig. 4. The findings revealed a statistically significant association between processed meat consumption and increased cancer risk, with a pooled hazard ratio of 1.19 (95% CI 1.15–1.25), indicating a 19% higher risk among processed meat consumers.

The analysis demonstrated moderate but significant between-study heterogeneity (*p* = 0.01), with an *I*2 value of 33%. The moderate heterogeneity likely reflects genuine differences in study populations, methodologies, or underlying biological mechanisms rather than chance variation alone.

The analysis assessing the relationship between processed meat consumption and colon cancer risk revealed evidence of a potential publication bias (Fig. 3D**)**. The funnel plot indicated asymmetry, a finding further supported by Egger’s test (intercept: 1.57, 95% CI 1.01–2.13, *t* = 5.485, *p* < 0.001). This suggests that smaller studies with non-significant results may be underrepresented in the dataset. In contrast, the investigation into colorectal cancer risk showed no indication of publication bias. The funnel plot appeared symmetric (Fig. 3E**)**, and Egger’s test confirmed the absence of significant asymmetry (intercept: 0.72, 95% CI − 0.27–1.72, *t* = 1.421, *p* = 0.166). These findings suggest that the overall conclusions regarding colorectal cancer risk are less likely to be influenced by selective reporting. Similarly, the analysis examining rectal cancer risk did not detect a publication bias (Fig. 3F**)**. The funnel plot displayed no discernible asymmetry, and Egger’s test corroborated this observation (intercept: 1.13, 95% CI − 0.02–2.28, *t* = 1.921, *p* = 0.084).

### Effect of total meat consumption

**Fig. 5:**
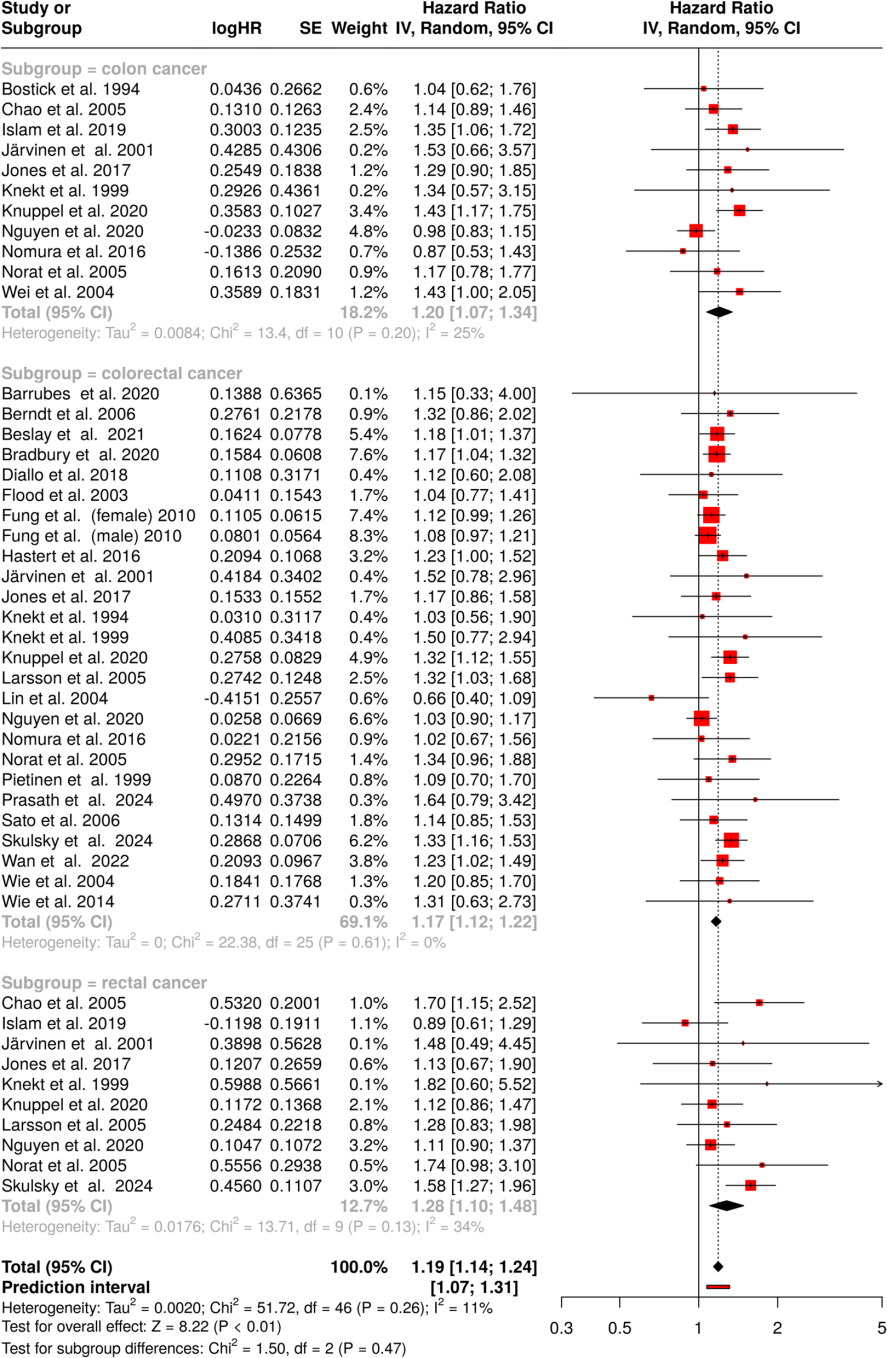
Forest plot illustrating the association between total meat consumption and cancer risk across different colorectal cancer subtypes. The diamond represents the pooled effect size for each subgroup and overall effect. The horizontal lines indicate the 95% confidence intervals (CI) for individual studies, with the size of squares proportional to study weight. The overall HR indicates an increased risk of cancer associated with total meat consumption (HR = 1.19, 95% CI 1.14–1.24). The analysis demonstrates moderate heterogeneity across studies (*I*^2^ = 11%; *p* = 0.26). *Abbreviations: CI*, confidence interval; *HR*, hazard ratio; *IV*, inverse variance; *SE*, standard error

A total of 47 cohorts were included in the meta-analysis to assess the association between total meat consumption and the risk of colon, colorectal, and rectal cancer (Fig. 5). The random-effects model using the inverse variance method calculated a pooled HR of 1.19 with a 95% CI of 1.14 to 1.24, indicating a statistically significant relationship and a 19% increase in the risk of cancer.

No significant heterogeneity was observed in the analysis across all included studies (*I*2 = 11%; *p* = 0.26). This moderate heterogeneity is likely a result of inherent variations in study populations, research methodologies, or biological mechanisms, rather than being due to random variations.

The evaluation of potential publication bias in the analysis of total meat consumption and its association with colon cancer did not reveal any evidence of bias. The funnel plot for colon cancer was symmetrical, suggesting no publication bias (Fig. 3G). This observation was supported by Egger’s test, which did not indicate funnel plot asymmetry (intercept: 0.45, 95% CI − 1.15–2.04, *t* = 0.551, *p* = 0.595). Similarly, the analysis of total meat consumption and the risk of colorectal cancer showed no signs of publication bias. The funnel plot was symmetrical, indicating the absence of significant bias (Fig. 3H). Egger’s test corroborated this finding by rejecting the presence of asymmetry (intercept: 0.21, 95% CI − 0.47 to 0.89, *t* = 0.601, *p* = 0.553). For rectal cancer, the analysis also demonstrated no evidence of publication bias. The funnel plot was symmetrical, further supporting the reliability of the included studies (Fig. 3I). This result was confirmed by Egger’s test, which did not detect any funnel plot asymmetry (intercept: 0.43, 95% CI − 1.36 to 2.22, *t* = 0.47, *p* = 0.651).

## Discussion

This meta-analysis provides strong evidence supporting the association between red and processed meat consumption and an increased risk of CRC, including both colon and rectal cancer. Our findings indicate that high consumption of red and processed meats is significantly associated with a greater risk of CRC, with hazard ratios suggesting an increased risk ranging from 13 to 22%, depending on the cancer subtype and type of meat consumed. These results are consistent with previous large-scale epidemiological studies and reinforce dietary recommendations advocating for the reduction of red and processed meat intake to lower CRC risk [6].

The biological mechanisms underlying this association are well-established. Processed meats, in particular, contain nitrates and nitrites, which can contribute to the formation of N-nitroso compounds (NOCs) [82–86]—carcinogens that promote DNA damage, oxidative stress, and inflammation in the colonic mucosa. Additionally, heme iron, which is abundant in red meat, plays a role in lipid peroxidation and may facilitate the formation of carcinogenic compounds in the gut [87, 88]. Another well-documented factor is the generation of heterocyclic amines (HCAs) [89–92] and polycyclic aromatic hydrocarbons (PAHs) [93–97] during high-temperature cooking, such as grilling or frying. These compounds have been shown to induce genetic mutations in colorectal epithelial cells, contributing to cancer initiation and progression. In addition to direct mutagens, trimethylamine N-oxide (TMAO) has recently emerged as a microbiota-derived metabolite implicated in CRC development [98]. TMAO is generated from dietary precursors such as carnitine and choline, abundant in red meat, through microbial metabolism in the gut [99–101]. Elevated circulating TMAO levels have been associated with increased CRC risk, potentially through mechanisms involving oxidative stress and chronic inflammation [98, 102–104]. These findings highlight the microbiome as a key intermediary in the relationship between red meat consumption and colorectal carcinogenesis, warranting further investigation into host–microbiota–diet interactions. The interplay of these mechanisms suggests that both the type of meat and the method of preparation are critical determinants of CRC risk.

Our findings align with previous systematic reviews and meta-analyses, yet they also address some of the inconsistencies in the literature. While prior studies have reported variable results, likely due to differences in study design, exposure assessment, and adjustments for confounders, our meta-analysis includes a comprehensive synthesis of prospective cohort studies, minimizing the risk of recall bias inherent in case–control studies. Furthermore, we conducted subgroup analyses, differentiating between colon and rectal cancers, providing a more granular understanding of how meat consumption may impact different segments of the colorectum. This distinction is particularly relevant given the emerging evidence that colorectal subsites may have distinct etiological pathways, influenced by dietary, microbiome, and genetic factors.

The public health implications of these findings are substantial. In the USA, red meat consumption remains significantly high, with the average American consuming 37 kg of beef per year, far exceeding the global average [105]. In 2021, total beef consumption reached approximately 13.6 million metric tons, the highest recorded amount between 2002 and 2023, with men and adults aged 20 to 49 being the largest consumers [106]. In comparison, Denmark had one of the highest per capita meat consumption rates globally, with the average person consuming 95 kg of meat per year in 2009, highlighting significant regional differences in dietary habits [107]. Additionally, 17% of 185 countries reported a daily intake of at least 100 g of unprocessed red meat, underscoring the global variability in red meat consumption patterns [107]. Given the widespread consumption of red and processed meats globally, even a modest increase in CRC risk translates into a considerable disease burden at the population level. The American Association for Cancer Research (AACR) recommends limiting red meat consumption to no more than three servings per week (approximately 350–500 g per week) and avoiding processed meat altogether [108]. Our results reinforce these recommendations and highlight the importance of dietary interventions as a feasible and effective strategy for CRC prevention.

CRC incidence and mortality vary widely across countries, influenced by genetic, environmental, and lifestyle factors, particularly dietary habits. Hungary stands out as a critical public health case, as it has one of the highest CRC incidence and mortality rates in Europe [109, 110], a burden that has been linked, in part, to historically high per capita red and processed meat consumption [107]. Despite the availability of CRC screening programs, participation remains suboptimal, contributing to late-stage diagnoses and poor survival outcomes. These challenges underscore the urgent need for targeted public health initiatives, particularly those aimed at promoting dietary modifications as a primary prevention strategy. To address these pressing public health concerns, the Semmelweis Study [111]—a large-scale, longitudinal cohort study—has been established to investigate the determinants of unhealthy aging in Hungary, with a particular focus on dietary patterns and lifestyle factors. Given Hungary’s high CRC incidence and mortality rates, the study aims to identify modifiable risk factors and provide a foundation for evidence-based interventions in CRC prevention. By integrating comprehensive dietary assessments, biomarker analyses, and epidemiological surveys, the Semmelweis Study evaluates the long-term effects of dietary modifications on health outcomes. Beyond observational research, the study informs intervention programs within workplaces and educational institutions to assess the feasibility and effectiveness of nutritional and lifestyle modifications in the Hungarian population.

In this context, limiting red meat consumption and encouraging adherence to a Mediterranean-style diet, which emphasizes fiber-rich foods, fruits, vegetables, whole grains, and healthy fats, may offer a protective effect against CRC [4]. The Mediterranean diet has been associated with reduced systemic inflammation, improved gut microbiota composition, and lower oxidative stress, all of which may counteract the carcinogenic effects of red and processed meat consumption. Beyond isolated dietary factors, emerging research on “ristoceutics” [112]—a concept referring to the combined use of protective functional foods—offers a promising avenue for dietary CRC prevention [113]. For example, the co-consumption of fiber-rich vegetables alongside red or processed meat has been shown to attenuate carcinogenic risks, potentially by binding mutagens or modulating gut microbiota [113–115]. A large cohort study from Alberta demonstrated that diets rich in vegetables, fruits, and whole grains may reduce the harmful effects of high meat intake on CRC risk [75]. Such findings support a shift toward dietary pattern-based recommendations, emphasizing food synergy and the cumulative effects of multiple components in modulating cancer risk [113]. Implementing dietary interventions along these lines alongside enhanced CRC screening efforts could be instrumental in reducing the national CRC burden in Hungary.

While this meta-analysis offers valuable insights, it is important to acknowledge certain limitations. The heterogeneity observed across studies is a key consideration, likely stemming from differences in dietary assessment methods, population demographics, and follow-up durations. Although all included studies were prospective in design, residual confounding remains a possibility, as factors such as physical activity, fiber intake, obesity, and gut microbiome composition may influence CRC risk. Additionally, self-reported dietary intake is inherently prone to measurement errors, and variations in meat processing methods across different populations may contribute to inconsistencies in risk estimation. Nevertheless, our use of a random-effects model and the subgroup analyses allowed us to mitigate some of these issues and enhance the robustness of our conclusions.

Increasing attention has also been paid to the role of genetic polymorphisms in modulating the impact of dietary risk factors such as red and processed meat [116–120]. Notably, individuals carrying the GSTM1 null genotype, who lack functional glutathione S-transferase M1 activity, may have reduced detoxification capacity, making them more susceptible to carcinogens derived from meat consumption [120]. Recent studies highlighted a significantly increased CRC risk in this genetically susceptible subgroup in relation to red meat intake, underscoring the importance of gene–diet interactions in colorectal carcinogenesis [120]. These findings suggest that precision nutrition strategies, tailored to individual genetic profiles, may enhance the effectiveness of CRC prevention efforts.

Future research should focus on clarifying potential effect modifiers that may influence the association between meat consumption and CRC risk. Studies investigating the impact of specific cooking methods, dietary interactions (such as fiber intake), and genetic predisposition could provide more precise risk estimates. Additionally, large-scale interventional trials assessing the effects of reducing meat intake on CRC incidence and gut microbiota composition would be valuable in strengthening causal inference. Emerging research on the role of gut microbiota in metabolizing dietary components suggests that the interplay between meat-derived carcinogens and microbial dysbiosis may be a crucial factor in CRC pathogenesis, warranting further investigation [121, 122].

In conclusion, this meta-analysis provides compelling evidence that high consumption of red and processed meats is significantly associated with an increased risk of CRC, colon cancer, and rectal cancer. These findings reinforce the importance of dietary modifications as a key strategy for CRC prevention and support existing public health recommendations advocating for the limitation of red meat intake and the avoidance of processed meats. Given the high CRC burden in Hungary and globally, urgent efforts are needed to promote evidence-based dietary guidelines, enhance CRC screening programs, and encourage lifestyle modifications that can help reduce the incidence of this preventable malignancy.
